# Antitumor Effects of Freeze-Dried Robusta Coffee (*Coffea canephora*) Extracts on Breast Cancer Cell Lines

**DOI:** 10.1155/2021/5572630

**Published:** 2021-05-18

**Authors:** Ayelén D. Nigra, Deborah de Almeida Bauer Guimarães, César G. Prucca, Otniel Freitas-Silva, Anderson J. Teodoro, Germán A. Gil

**Affiliations:** ^1^Departamento de Química Biológica Ranwel Caputto, Facultad de Ciencias Químicas, Universidad Nacional de Córdoba-CIQUIBIC, CONICET, Córdoba, Argentina; ^2^Universidade Federal do Estado do Rio de Janeiro, Laboratory of Functional Foods, Rio de Janeiro, CEP 22290-240, Brazil; ^3^Empresa Brasileira de Pesquisa Agropecuária-Embrapa Agroindústria de Alimentos, Av. das Américas 29.501, Rio de Janeiro–RJ 23020-470, Brazil

## Abstract

Coffee consumption is believed to have chemopreventive and chemotherapeutic effects and to contribute to preventing the development and progression of cancer. However, there is still controversy around these claims. As indicated in our previous works, diet can influence the risk of breast cancer. Intake of coffee is hypothesized to reduce this risk, but current scientific evidence is not conclusive. This work is aimed at studying the effects of Robusta coffee bean extract on cell viability, proliferation, and apoptosis of different human cancers, especially breast cancer cell lines. To this end, cell viability was evaluated by Alamar Blue in 2D and 3D models, the cell cycle by PI, apoptosis by annexin V, mitochondrial morphology, and functionality by mitoTracker, and colony formation capacity by the clonogenic assay. Green and dark coffee extract significantly reduced viability in human breast, colorectal, brain, and bone cancer cells. Coffee anticancer activity was clearly evidenced in MDA-MB-231 (ER^−^) and MCF-7 (ER^+^) breast cancer cells but not in the normal breast cell line. In addition, coffee extract induces an increase S phase and a decrease G2/M population in breast cancer cells, affected the mitochondrial morphology, and triggered apoptosis. MDA-MB-231 breast cancer cells lost their clonogenic capacity after treatment. The antitumor activity was demonstrated in both 2D and 3D culture cell models.

## 1. Introduction

Cancer is currently a major public health problem, and the available therapeutic strategies are not fully effective in several tumor types. The American Cancer Society estimates that by 2019, approximately 17,624,50 new cancer cases will be diagnosed, which is equivalent to more than 4,800 new cases every day [1] In addition, there will be approximately 268,600 new female breast carcinoma cases in the United States [2]. Among the different cancer types, breast cancer is one of the most frequently diagnosed and the leading cause of cancer death in females worldwide.

Breast cancer etiology is considered multifactorial, and it includes interactions between genetic, behavioral, and environmental factors. Breast cancer is a heterogeneous disease, but cancer subtypes are hormone-related. Breast tumors that express the ER (ER^+^ tumors) are more strongly associated with hormone-related factors than tumors that do not express the ER (ER^−^ tumors).

According to an analysis that verified the antitumor molecules approved by drug entities, such as FDA and similar organizations, 49% of anticancer molecules approved between 1940 and 2014 were natural products or chemical derivatives worldwide [3]. To understand the action of promising molecules in cancer treatment, it is necessary to characterize active chemical components and study plants in vitro and animals before clinical studies are conducted. Phytochemicals, due to their dietary origin, are considered safe, well-tolerated, and low-toxicity compounds, enabling the synthesis of semisynthetic medicinal agents [4].

As stated in previous work, the diet may influence breast cancer risk. Coffee intake has been hypothesized to reduce the risk of breast cancer, but the current evidence is inconclusive [5, 6]. Coffee is the second most popular beverage in the world after water, and due to its low cost and ease of preparation, it is consumed in almost all countries and by all social classes of the population through different preparation modes. Coffee is a complex beverage that contains hundreds of biologically active compounds and exerts potent effects on long-term human health. A large number of recent studies have focused on health outcomes associated with coffee intake [7].

This drink contains caffeine, chlorogenic acid (CGA) (which is caffeic bound to quinic acid), p-coumaroylquinic, and feruloylquinic acids [8]. Although these acids are widely present in beverages made from herbs, fruits, and vegetables, consumption of these drinks represents only 5-10% of coffee consumption. Coffee roasting is an important step during coffee preparation, which improves its sensory properties. In a previous study, we analyzed the coffee compounds present in the extracts and observed that the roast process led to a significant reduction in all of them [9]. This could be explained by both amino acids and reducing sugars acting as a substrate in the Maillard reaction. In the roast, CGAs are hydrolyzed and new products, such as lactones, are formed, and this process changes the antioxidant profile of green coffee [10]. Moon et al. [11] reported that up to 99% of CGA can be lost with the highest roasting [11].

Green coffee consumption as a dietary supplement or as a beverage is increasing due to its reported antioxidant benefits. Furthermore, the chemopreventive and anticancer potential of bioactive molecules present in a standard cup of coffee has been described not only in green coffee but also in the beverage made from coffee, the black coffee. Indeed, many retrospective meta-analysis and several human studies have shown the benefits of coffee consumption for reduced the incidence and risk of breast cancer (without showing possible mechanisms) ([12–16], Vatten and Løken 1990, [17–20]), while some in vitro studies have shown that coffee induces antiproliferative effects in breast cancer cells MDA-MB-231 [21]. Despite the information available, more research is needed to understand the benefits of coffee. The aim of this study was to investigate this knowledge gap, analyzing the effect of Robusta coffee bean extracts obtained by freeze-drying after different roasting processes and their anticancerogenic effects both in 2D and 3D models on breast carcinoma cell lines.

## 2. Materials and Methods

### 2.1. Coffee Samples

The coffee used in this study was *Coffea canephora*. The green coffee beans were purchased from coffee producers in Colatina-Espírito Santo, Brazil. The coffee bags were transported to Rio de Janeiro and stored at the Laboratory of Molecular Diagnostics and Mycology and then processed in EMBRAPA Food Technology, Rio de Janeiro, Brazil. Green coffee beans were selected after the elimination of dirt and defective beans. Part of the green grains was milled in an analytical grinder (IKA®A11 basic) to produce the green coffee solution, and the remaining beans were roasted in a grain roaster (Gene Café®). Dark roast was performed at 245°C for 15 min followed by Agtron scale. Then, they were milled in a homemade grinder (Cuisinart®) and an analytical grinder (IKA®A11 basic). Coffee powders were sieved through an analytical sieve (710 *μ*m). The extracts at 50% w/v were prepared in hot water (90-95°C) for 10 min. To optimize the extraction procedure, ultrasound with sonotrode (Hielscher®UIP1000hdT) and an ice bath (216 to 60 W; *A* = 70% for 10 min) were performed. Then, the solutions were centrifuged at 5643 RCF/5 min (Rotina 38R–Hettich zentrifugen), and the supernatant was dried in a freeze-drier (dehydration at 60°C; air velocity of 1 m/s for 32 h). The dehydrated extracts were stored in vacuum-laminated zip-type packages at -80°C until analysis. Each 300 mL of 50% coffee solution yielded 30 g of dry extract.

### 2.2. Cell Culture and Treatment Protocol

Human breast carcinoma (MCF7 and MDA-MB-231), human breast (MCF 10A), human bone carcinoma (U2OS), human colorectal carcinoma (HCT116), and human brain glioblastoma multiform (T98G) cell lines were obtained from the ATCC-Bethesda, MD, USA. Cell lines were plated and maintained routinely in Dulbecco's Modified Eagle's Medium high glucose (DMEM) supplemented with 10% fetal bovine serum (FBS) plus antibiotics (100 U/mL penicillin/0.1 mg/mL streptomycin), and pH 7.4, under 5% CO_2_. Culture medium for MCF 10A cell line was supplemented with 20 ng/mL epidermal growth factor, 100 ng/mL cholera toxin, 0.01 mg/mL human insulin, and 500 ng/mL hydrocortisone. Once the cells reached 80% confluence, they were dissociated using 0.05% trypsin-EDTA and subcultured. Culture medium was replaced every 2 days. Cells were seeded in 96-well plates, and after 24 h, the medium was changed to fresh supplemented DMEM medium. Cells were treated for 24 h with increasing concentrations of green or dark coffee extracts dissolved in supplemented DMEM (25 to 5000 *μ*g/mL). Untreated cells were included in each plate. Subsequently, a cell proliferation assay was performed.

### 2.3. Cell Viability Assay

Cell lines were plated in 96-wells plates and cultured for 24 h. Then, cells were treated for 24 h, and the culture medium replaced with alamarBlue® 10% v/v dissolved in DMEM supplemented with 10% FBS and antibiotics. Three hours later, fluorescence (590 nm) was monitored using a Biotek microplate reader, as recommended by the manufacturer.

### 2.4. Cell Cycle Analysis

After treatment, cells were rinsed briefly with phosphate buffered saline (PBS) and detached using trypsin at room temperature. After centrifugation, the cells were washed twice with PBS, resuspended in cold 70% v/v ethanol solution, incubated for 24 h at 4°C, and treated with RNAse (200 *μ*g/mL) for 30 min at 37°C. Then, the cells were stained using propidium iodide (PI) (50 *μ*g/mL). The cell suspension was analyzed for DNA content by flow cytometry using a Beckton Dickinson FACSCanto II flow cytometer. The relative proportions of cells with DNA content indicative of apoptosis (<2*n*), G0/G1 diploid (2*n*), S (phase > 2*n* but < 4*n*), and G2/M phase (4*n*) were determined using FlowJo V10 software (10.7.1). Cell cycle analyses were performed by the Watson Pragmatic algorithm model. Nuclei of viable cells were gated according to the SCC-H × FL2-H ratio [22].

### 2.5. Apoptosis Analysis

To measure the apoptosis rate, the cells were stained using FITC-conjugated Annexin V and PI. The nonadherent cells were collected, and the adherent cells were quickly washed with PBS and detached using trypsin/EDTA 0.125% (Sigma chemical Co., St. Louis, USA) at room temperature. Subsequently, cells were stained with Annexin V-FITC/propidium iodide (PI) (BD Pharmingen, New Jersey, USA) according to the manufacturer's instructions, quantified by flow cytometer using a Beckton Dickinson FACSCanto II and analyzed using FlowJo V10 software [9].

### 2.6. Colony-Forming Units

Exponentially growing MDA-MB-231 and MCF 10A cells were harvested, counted, and seeded (1.10^6^ cells/plate) in Petri dishes. Cells were allowed to grow at 37°C in 5% CO_2_ overnight. Then, cells were incubated with different coffee concentrations for 24 h. Next, the cells were harvested, counted, and reseeded at low density (about 50-250 cell/well) in 24 multiwell plates (Corning Costar USA). After incubation for additional 15 days, the colonies were stained with crystal violet 5% v/v solution for 20 min. The number of clones (colonies > 50 cells) in a given area was counted for each condition [23].

MCF7 is a breast ductal carcinoma cell line that corresponds to a relatively less aggressive hormone-responsive breast cancer (better prognosis). MCF-7 cells express lower levels of VEGF than MDA-MB-231 cells, which have high invasive and migration capacities. Due to this, we believe that MCF7 cell lines were not suitable for the colony formation assay [24, 25].

### 2.7. Mitochondrial Morphology

MDA-MB-231, MCF7, and MCF 10A cells were grown on 12 mm glass coverslips and treated using coffee extracts (0 and 1000 *μ*g/mL) for 24 h. Next, cells were incubated with 100 nM MitoTracker Red CMXRos (Invitrogen M7512), dissolved in DMEM, for 30 min at 37°C and washed twice with PBS [26]. The cells were then fixed using 4% paraformaldehyde solution for 15 min at room temperature, washed twice with PBS, and mounted using Fluorsafe (Calbiochem). Images were collected using a confocal microscope Olympus FV1200 (Super-corrected 60X objective–plapon 60XOSC).

### 2.8. Mitochondrial Functionality (MMP)

MDA-MB-231, MCF7, and MCF 10A cells were grown on 24 multiwell plates for 48 h and treated using coffee extracts (5000 and 1000 *μ*g/mL) for 4 and 24 h. Next, cells were collected by trypsinization and incubated with 100 nM MitoTracker Red CMXRos (Invitrogen M7512) for 30 min and then analyzed by flow cytometry in a Forteza cytometer. The frequency histogram analysis was performed using FlowJo V10 software.

### 2.9. Spheroid Generation

The spheres were formed from cells using the hanging drop system: Perfecta3D® 96-well plates (3D Biomatrix; Michigan, USES) 6 × 10^3^ for MDA-MB-231 or MCF 10A and suspended on the lid of a Petri dish for MCF7. Partial medium exchange was made every two days. After incubation of the MDA-MB-231 cell line for 7 days, MCF7 cell line for 6 days, and MCF 10A cell line for 4 days, the spheroids were treated with coffee extracts at the desired concentration for 24 h. After treatment, the spheres were observed using a Leica microscope and then incubated with alamarBlue ® 10% v/v in DMEM supplemented with 10% FBS and antibiotics for 24 h. The plate was read using the Biotek plate reader to measure the fluorescence at 590 nm [27].

### 2.10. Statistical Analysis

Results are expressed as mean values and the corresponding standard deviation of experiments done in triplicate. The data were analyzed with the statistical software GraphPad Prism (version 8.0, GraphPad software, San Diego, CA). The significant differences are indicated by letters as determined by one-way analysis of variance (ANOVA) followed by Tukey's posttest (*p* < 0.05).

## 3. Results and Discussion

The coffee extracts used in this study were obtained by standardized processes by the Brazilian national company of “Pesquisa Agropecuária” from green *Coffea canephora* beans acquired from coffee producers in Colatina-Espírito Santo, Brazil. They were carefully selected, transported, ground, roasted, sieved, lyophilized, and fried using standardized times and temperatures to ensure reproducible results. Robusta green and roasted coffee extracts made in this study were obtained as close as possible to the typical preparation of a coffee for consumption (See Section 2 for further information).

**Figure 1:**
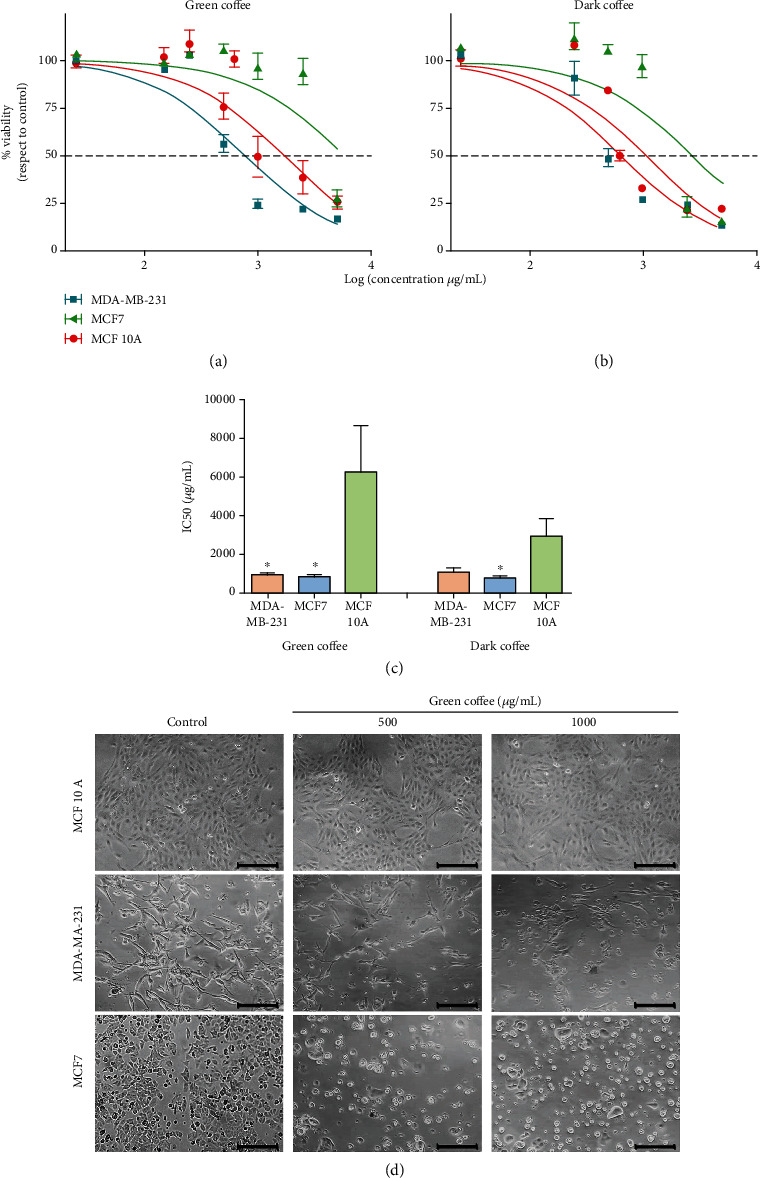
Coffee extract effect on cell cancer viability. Human cancer cell lines (MCF7 and MDA-MB-231) and human epithelial breast cell lines (MCF 10A) were treated for 24 h with increasing concentrations of (a) green coffee or (b) dark coffee (0-5000 *μ*g/mL). (c) Inhibitory concentration 50% (IC50) on breast cell line treated with green and dark coffee was determined and expressed as *μ*g/mL. ^∗^ represents statistically significant difference in the column indicated with respect to treated MCF 10A, as determined by ANOVA (*p* < 0.05), with Tukey's multiple comparison posttest. (d) Cell morphology was analyzed by light microscopy. Scale bar = 50 *μ*m. Values are represented as mean ± SEM from three independent experiments.

Experiments were carried out to evaluate the effects of coffee extracts on cancer cells. For this purpose, cell viability was analyzed after incubation with different concentrations of coffee extracts dissolved in DMEM medium for 24 h. As observed in Figures 1(a) and 1(b), human cancer cell line viability (breast carcinoma) was significantly decreased in a concentration-dependent manner. Surprisingly, a comparable effect was observed in cancer lines from colon (CHT116), brain (T98G), and bone (U2OS) (Supplementary Figure 1A-C). Our data show that both green and dark coffee extracts inhibited the proliferation of MCF7 (ER^+^) and MDA-MB-231 (ER^−^) human breast cancer cell lines (Figures 1(a) and 1(b)). However, cell viability was not affected in the breast nontumorigenic MCF 10A cell line except at high concentration (log 3 *μ*g/mL = 1000 *μ*g/mL) (Figure 1(c)). Several coffee constituents may differentially affect ER^+^ and ER^−^ breast cancer subtypes. Rosendahl et al. [28] showed that caffeine doses significantly inhibited the proliferation and total cell number of both ER^+^ MCF7 and ER^−^ MDA-MB-231 breast cancer cells, with a maximum inhibition at 5 mmol/L. However, caffeine alone is not able to match the effects of coffee [28].

The chemical composition of coffee varies because the preparation process may change the content of bioactive compounds, resulting in different types of coffee beverages around the world, as widely reported in the literature. During roasting, the chlorogenic acid content decreases, while melanoidins are formed as complexes of sugars, amino acids, and chlorogenic acid through the Maillard reaction, or vitamin B niacin from the trigonelline alkaloid. Instant coffee or a paper filter on the coffee beans leads to almost complete removal of the diterpenes of cafestol and kahweol. The caffeine content varies according to the type of coffee and can also be modified by the preparation process [29]. Recent studies have described the presence of phytochemicals with proven bioactive effects in coffee, such as caffeine, polyphenols, trigonelline, caffeic acid, melanoidins, acid nicotinic, flavonoids, CGAs, kahweol, and cafestol [30–32] and the health benefits of these compounds. Nevertheless, the mechanisms responsible for the chemopreventive coffee effects continue to be studied for a better understanding. Various health benefits of coffee have recently been studied, with special emphasis on its protective effect against DNA damage. Our previous publication demonstrated that coffee has an important activity and antioxidant capacities in prostate cell cancer, decreasing the cell proliferation and inducing programmed death in apoptosis [9]. Therefore, coffee can be used as a chemopreventive and chemotherapeutic agent that contributes to preventing cancer development and progression [30, 31, 33, 34]. The bioactive components of coffee on the human body affect the endogenous antioxidant and detoxification processes [35]. In addition, coffee has a suppressive effect on the proinflammatory signaling pathway and on the carcinogenesis process itself. The coffee components interfere with all carcinogenesis phases: initiation, progression, and metastasis [29].

Caffeine and the polyphenol content have been suggested to contribute to coffee anticancer activities. Dried green coffee beans contain carbohydrates (59–62%), CGAs (7–10%), aliphatic acids (2%), caffeine (1–2%), trigonelline (1%), and free amino acids (<1%), but roasting coffee reduces the contents of carbohydrates, CGAs, and free amino acids and increases those of alkaloids (mostly caffeine) and aliphatic acids [36]. Although, theoretically, the bioactive compound content is lower in roasted coffee, due to the high-temperature process, we tested cell viability after treatment with dark roasted coffee extracts, since the consumption of roasted coffee is greater in the diet. Although both types of coffee showed a significant antitumor effect on both MDA-MB-231 (ER^−^) and MCF7 (ER^+^) breast cancer cell lines, the green coffee extract showed a lower cytotoxic effect on MCF 10A cells (compared to dark coffee), and therefore, it was selected to continue the studies.

To investigate the effects on cell morphology, breast cancer cell lines were incubated with green coffee extracts at concentrations of 500 and 1000 *μ*g/mL for 24 h. As shown in Figure 1, MDA-MB-231 and MCF7 cell morphology was significantly altered showing rounded cells, which indicates cell detachment and death. Besides, green coffee decreased cell density but did not alter cell density and morphology in MCF 10A cells (Figure 1(d)). STS was used as a positive control of apoptotic damage (a well-known apoptotic inducer) [37].

**Figure 2:**
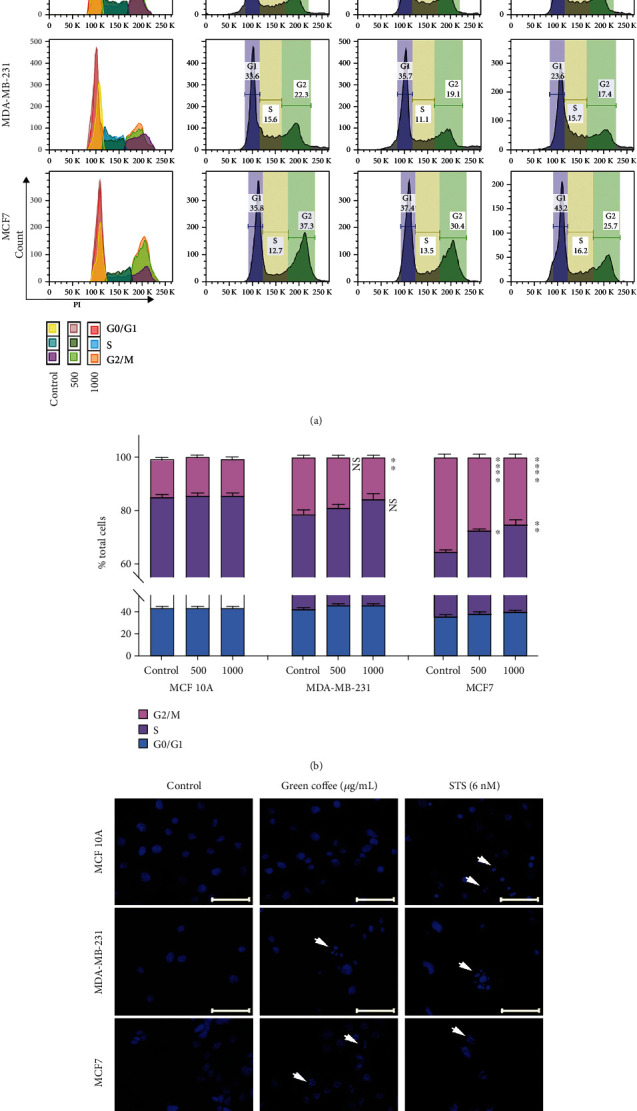
Coffee extract effect on cell morphology and cell cycle progression. MCF 10A, MDA-MB-231, and MCF7 cells were treated during 24 h with green coffee extract at 0 (control), 500, or 1000 *μ*g/mL. (a) DNA content profiles using propidium iodide stain to compare the cell cycle progression. (b) Quantification of cell population proportion in different cell cycle phases. Values represent the mean ± SEM from three independent experiments. Cell cycle were determined using FlowJo V10 software (10.7.1) and analyzed by the Watson Pragmatic algorithm model. Each concentration of coffee extract used was compared with its respective cell line control (^∗^*p* < 0.005; ^∗∗^*p* < 0.001; ^∗∗∗^*p* = 0.0001) as determined by ANOVA, with Tukey's multiple comparison posttest. (c) Nuclear fragmentation was analyzed using Höestch 33342 staining. Staurosporine (STS) was used as a positive control for nuclear fragmentation. The white arrows indicate fragmented nuclei. Scale bar = 50 *μ*m.

The results of the cell cycle analysis indicate that the treatment of cells with green coffee extract at 1000 *μ*g/mL led to an increase S-phase and a decrease G2/M population of the cell cycle in both breast cancer cell lines (MDA-MB-231 and MCF7). In contrast, it was not observed modifications in the cell cycle population distribution of MCF 10A normal breast cell line, with the same treated (Figures 2(a) and 2(b)). No previous work has described the effects of coffee extract on cell cycle arrest in ER^−^ breast cancer cell lines.

**Figure 3:**
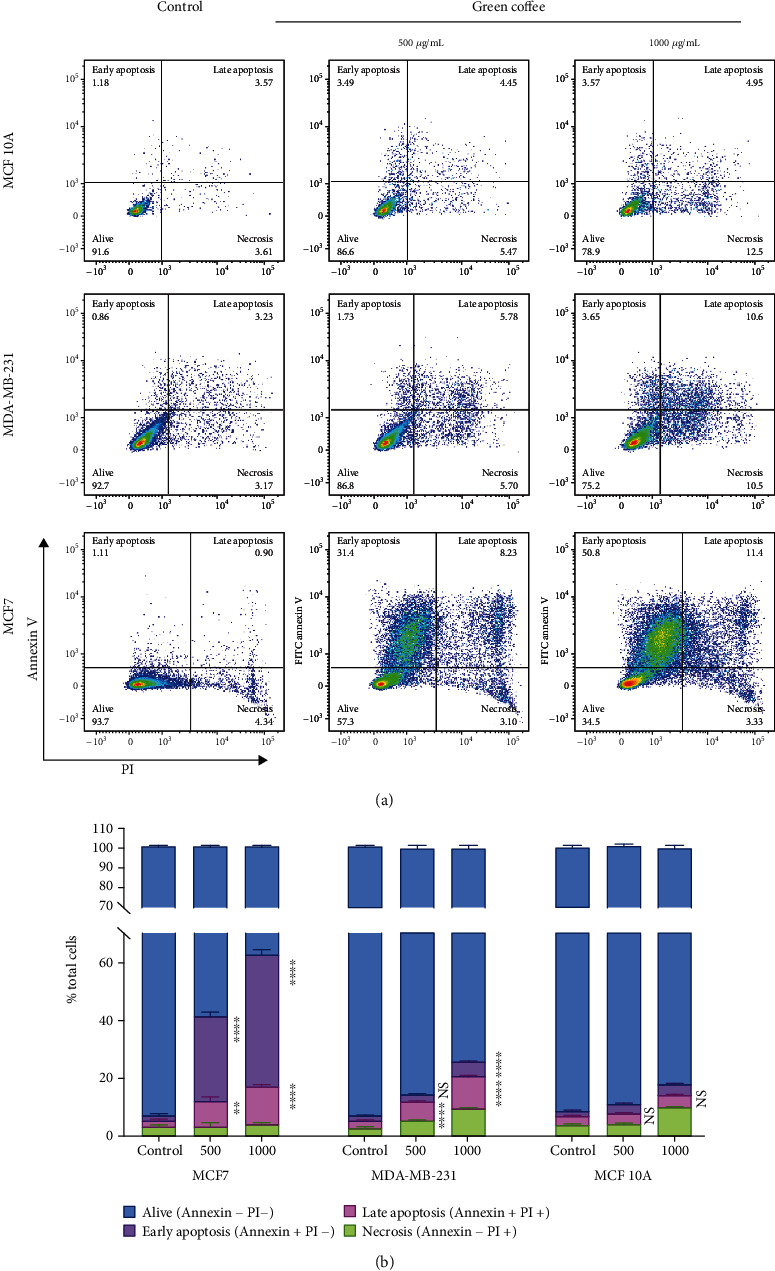
Apoptosis induction by green coffee extract. MCF 10A, MDA-MB-231, and MCF7 cells were treated for 24 h with green coffee at 0, 500, or 1000 *μ*g/mL. (a) Apoptosis flow cytometry analysis by Annexin V/propidium iodide staining was performed, and representative plots are presented. (b) Quantification of apoptosis induction (relative to control). Values are mean ± SEM from three independent experiments (^∗∗∗^*p* < 0.0001) as determined by ANOVA, with Tukey's multiple comparison posttest.

Our results show that cell incubation with coffee extracts induces cell death. Consequently, we next evaluated cell death type triggered by nuclear fragmentation analysis. After treatment, cells were stained using Höechst 33342, and nuclear morphology was analyzed by fluorescent microscopy. A chromatin condensation distinctive pattern and nuclear fragmentation (apoptotic bodies) were observed in the MDA-MB-231 and MCF7 cells treated, but not in MCF 10A cells (Figure 2(c)). Next, we also analyzed cell death type triggered by green coffee extracts using propidium iodide/Annexin V stain by a cytometric assay. Figures 3(a) and 3(b) show the percentage of apoptosis induction by coffee extracts, in MCF7 and MDA-MB-231 breast cancer cell lines (60 and 15% induction, respectively). In contrast, coffee extract had no apoptotic effect on MCF10A breast nontumor cell lines, showing less of 8% apoptotic cells.

The antitumor effect of coffee is thought to be based on several mechanisms. The antioxidant effect is conditioned not only by the content of direct antioxidants but also by the ability to activate endogenous antioxidant enzymes like superoxide dismutase (SOD) and *γ*-glutamylcysteine synthetase [38, 39]. At present, substantial experimental evidence shows that it is possible to prevent mitochondrial damage by protecting against oxidative stress [40]. Furthermore, mitochondrial oxidative stress plays a key role in apoptosis [41]. In different cell types, mitochondrial fragmentation induced by oxidative stress has been reported [42, 43]. Additionally, the process of apoptosis initiation is associated with mitochondrial fragmentation and the release of proapoptotic proteins [44, 45]. Therefore, we examined the effects of green coffee extracts on mitochondrial morphology in MDA-MB-231, MCF7, and MCF 10A cells.

**Figure 4:**
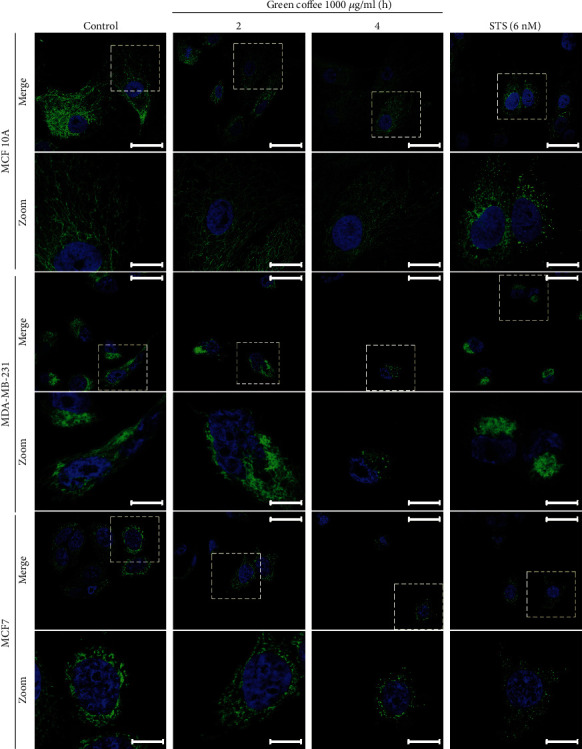
Green coffee effect on mitochondrial morphology. MDA-MB-231, MCF7, and MCF 10A cells were treated for 0, 2, and 4 h with green coffee extracts at 1000 *μ*g/mL or STS 6 nM for 4 h. Representative microphotographs show the mitochondrial (green-MitoTracker Red CMXRos) and nuclear (blue-DAPI) stain. Scale bar = 50 *μ*m (upper row), zoom scale bar = 10 *μ*m (lower row).

Mitochondria are critical organelles that ensure the normal function of the cells. These organelles play a crucial role in generating cellular energy and regulating cell fate through their participation in cell-death regulation [46]. Mitochondria morphology was analyzed using the mitochondrion-selective probe Mitotracker Red CMXRos, and, as illustrated in Figure 4, green coffee extracts induced important changes and fragmentation in MDA-MB-231 and MCF7 mitochondria, observed as dotted (3^rd^ column). Normal mitochondrial morphology (as observed in control cells, 1^st^ column) was altered after 4 h of treatment with green coffee, while MCF 10A cells maintained their morphology intact.

**Figure 5:**
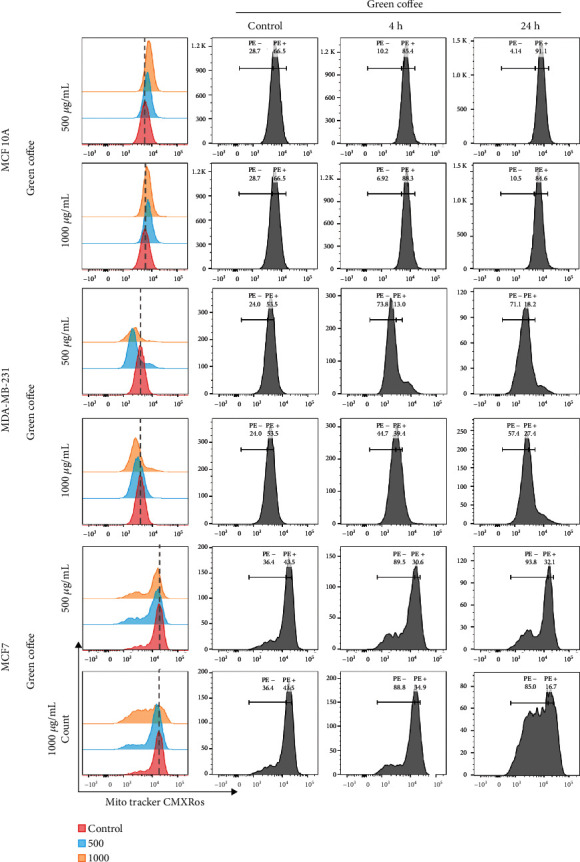
Green coffee effect on mitochondrial membrane potential. MDA-MB-231, MCF7, and MCF 10A cells were treated for 0, 4, and 24 h with green coffee extracts at 500 and 1000 *μ*g/mL. Cells were collected and incubated with 100 nM MitoTracker Red CMXRos for 30 min and then analyzed by flow cytometry. Representative histograms of each treatment are presented.

Since changes in mitochondrial membrane potential (MMP) are associated with cell death and given the results of the microscopy analysis (Figure 4), in which we observed a considerable change in mitochondrial morphology related to the incubation with coffee extracts, we analyzed the MMP using MitoTracker Red CMXRos stain and flow cytometry. The accumulation of this probe depends on the MMP, and therefore, a diminution in its accumulation is associated with changes in the MMP [47]. To evaluate this process, MCF10 A, MCF7, and MDA-231 cells were incubated for 4 and 24 h in the presence of 500 or 1000 *μ*g/mL of coffee extracts. Cells were then collected by trypsinization, stained using MitoTracker Red CMXRos, and then analyzed by flow cytometry. As shown in Figure 5, the incubation of cells with the extract for 4 or 24 h triggered a significant reduction in the probe accumulation compared to that observed in control (show as a shift of the peak towards the left side, with respect to the control of each cell line). This reduction in probe accumulation, related to MMP changes, was observed in both malignant breast cancer cell lines, but not in MCF10 A, in which the MMP sensitive probe accumulated similarly in the presence or absence of coffee extracts.

In agreement with our results, several studies proposed that caffeine treatment significantly suppressed cell growth and viability and induced apoptosis by activating the caspase pathway in gastric cancer cells [48], epidermal cells [49], and glioma cells [50]. Also, Furtado et al. noted that coffee and caffeine protect the liver from thioacetamide-induced injury in rats by reducing cleaved caspase-3 labeling indexes and growth fraction [51]. Caspase-3 is involved in apoptosis induction. It mediates the proteolytic cleavage of key proteins, such as the nuclear PARP, which plays a role in chromatin condensation and degradation in apoptotic cells [52]. However, the function of caspase-3 in the pathway of coffee-induced apoptosis is not clear [49]. It was observed that the treatment with coffee extracts induces activation of caspase-3 in MDA-231-MB cell line (Supplementary Figure 1D-E) suggesting that apoptosis signaling mediated by coffee extract triggered cell death and involves the cleavage of this cysteine protease.

**Figure 6:**
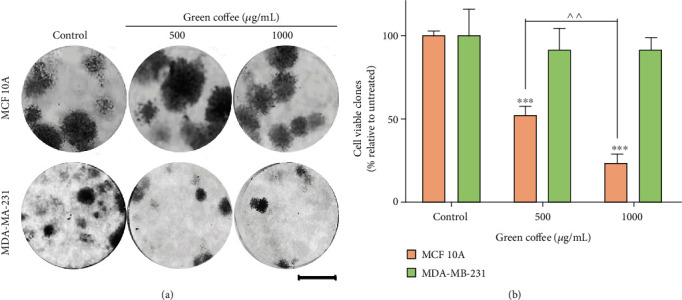
Green coffee effect on MDA-MB-231 and MCF 10A cell clonogenic capacity. Cells were treated for 24 h with green coffee extracts at 0, 500, and 1000 *μ*g/mL. Then, the surviving cells were seeded at low density for a clonogenic assay. (a) Representative results are shown for each condition stained with crystal violet. Scale bar = 0.5 cm. (b) Quantification of the viable clones. Values are the mean ± SEM of three independent experiments (^∗∗∗^*p* = 0.0001 with respect to control and ^^*p* < 0.001) between cases as determined by ANOVA, with Tukey's multiple comparison posttest.

To further investigate the effect of coffee on breast cancer cells, we conducted a colony formation assay (in vitro cell survival assay, based on the single-cell ability to grow in a colony). This assay allowed us to determine the cell clonogenic capacity after exposure to cytotoxic agents [53]. The results showed that green coffee extracts significantly inhibited the number of colonies formed by MDA-MB-231 cells after treatment, relative to untreated cells, thus acting as a cytotoxic and/or cytostatic agent. Additionally, we observed that the treatment did not affect MCF 10A cells' clonogenic capacity (Figure 6). Although the assay was repeated by increasing the number of cells to 500/well, MCF7 was unable to form colonies under controlled conditions. Due to their ability to grow into domes, MCF7 cells were not suitable for the colony formation assay [24, 25].

Our results are similar to those found in prostate tumor cells (DU-145) treated with coffee CGAs, which showed a reduced colony formation capacity after treatment [54]. Besides, anchorage studies showed that CGAs can form stable complexes with protein kinase and induce this protein translocation from cytosol to the plasma membrane, triggering cell death via disruption of mitochondria [55]. It was observed that 5-CQA in breast cancer cell line MDA-MB-231 (a CGAs: 5-O-caffeoylquinic acid) can modulate the activity of Ras proteins by inhibition upon binding to the target [56]. Further research is needed to elucidate the molecular basis of the activity of natural extracts of green and roasted coffee beans as chemoprotective agents reported in this work.

**Figure 7:**
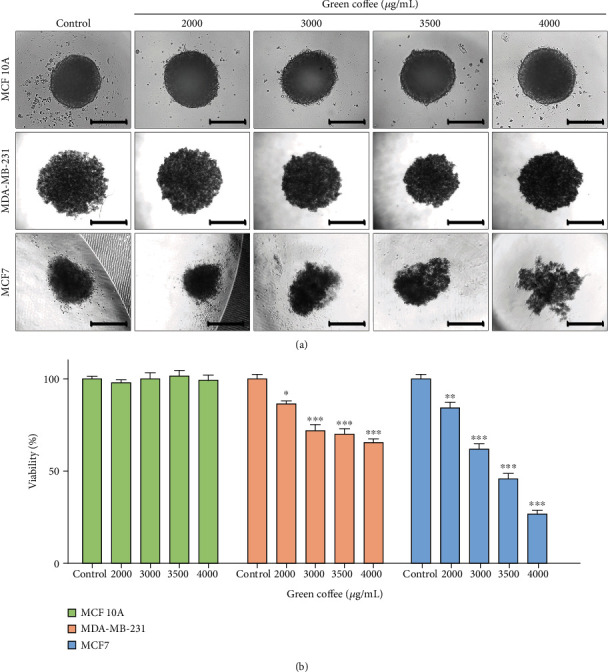
Green coffee extract effect on a 3D model of spheroids. 6 × 10^3^ MDA-MB-231, MCF7, or MCF 10A cells were seeded (40 *μ*L) and cultured for 7, 6, and 4 days, respectively. Spheroids were treated with green coffee extracts at 0, 2000, 3000, 3500, and 4000 *μ*g/mL for 24 h. (a) Representative microphotographs showing spheroid morphology are presented. Scale bar = 100 *μ*m. (b) Spheroids viability was analyzed for the AlamarBlue ® assay and quantified. Values are the mean ± SEM from three independent experiments (^∗^*p* < 0.005; ^∗∗^*p* < 0.001; ^∗∗∗^*p* = 0.0001) between cases as determined by ANOVA, with Tukey's multiple comparison posttest.

In addition, we investigated the therapeutic potential of green coffee extract on a 3D cell culture model, generating spheroids from MCF 10A, MCF7, and MDA-MB-231 cells. On days 4, 6, or 7 of culture, respectively, these spheroids were treated with different concentrations of green coffee extracts for 24 h and then analyzed by microscopy. MDA-MB-231 and MCF 10A spheroid morphology was not modified by the treatment, but MCF7 spheroids lost their cellular compaction level until forming very lax aggregates (Figure 7(a)). A significant decrease in cellular viability of MDA-MB-231 and MCF7 spheroids was observed. Despite this strong anticancer effect, the viability of MCF 10A did not show alterations (Figure 7). As previously reported [57], we observed that spheroids were resistant to a higher cytotoxic agent concentration in comparison with cells grown in monolayers.

The ability of dietary substances to modulate the immune response and suppress the proinflammatory environment in the body is considered to be one of the important mechanisms of tumor chemoprevention [35]. In humans, after food consumption, the level of each nutraceutical in the blood plasma varies, making it even more difficult to attribute the possible anticancer effects of coffee to a specific molecule. In fact, only a small percentage of the ingested compounds enter the circulatory system and reach tissues, and very little of the absorbed material retains the original structure present in the beverage [31]. In this work, the treatment of 1000 *μ*g/mL coffee extract is equivalent to 0.001 g coffee dry extract (powder), which represents 0.05 mL of coffee ready to drink in a 10% solution (this is a common concentration of daily coffee consumption). Several constituent compounds of coffee have important antioxidant activities. Ingredients other than caffeine, such as CGAs and caffeic acid, are antioxidant in nature, and their presence slows down the inflammation process, thereby providing protection from the hazardous effect of free radicals and from endothelial damage. In fact, coffee intensifies the antioxidant defense mechanism of the immune system by inducing the expression of mRNA and enzymes mitigating the negative effects of free radicals on neurodegeneration [30]. Caffeine has been positioned as a protective agent for cell membranes against oxidative damage, with anticancer activity [33], and CGAs (the most important class of polyphenols) are well known as powerful antioxidants [31, 34].

In previous work, the antioxidant activity of extracts with different degrees of roasting was analyzed. We demonstrated, through several tests, that Robusta green coffee and lightly roasted extracts had a higher antioxidant potential [9]. In this work, we document the stimulation of mitochondrial fragmentation by coffee in breast cancer cells, possibly induced by oxidative stress, which was not observed in noncancer breast cells.

A reduction or even elimination of coffee consumption has been traditionally recommended in view of a global risk profile, but its consumption has progressively been considered in a less negative light due to its better-known phytochemistry [58]. The knowledge that coffee and caffeine are not equivalent has increased the interest in determining whether other components of coffee might contribute to the protective action in the human body. Coffee is a complex beverage containing more than 1000 described phytochemicals responsible for its pleasant flavor, aroma, and health promoters. Several recent scientific publications have described beneficial coffee effects and discussed potential mechanisms of action, including anti-inflammatory [59, 60], antiamyloidogenic [61], antimicrobial, antiglycative [62], hepatoprotective, antidiabetic, and antiosteoclastogenic effects [63, 64].

Recent epidemiological and prospective (meta-analysis) studies demonstrate that consumption of healthy foods, such as coffee, especially rich in polyphenol content, might have antitumor activity against several cancer types, such as colorectal [65], nonmelanoma [66], endometrial [67, 68], esophageal [69], pancreatic [70], and prostate [71].

Especially in reference to breast cancer, numerous authors provided evidence on a strong and significant inverse association between cancer risk and coffee consumption [16], including carrying the BRCA1 mutation subgroup of patients [72], with ER^−^ tumors [13, 14], tamoxifen-treated with ER^+^ tumors [15], and postmenopausal tumors [12]. Some studies have shown that women who consume regular coffee had 20 to 50% reduction in the incidence of breast cancer [19, 73]. Other authors report the metastasis decrease [18, 20] and breast cancer cell apoptosis induction. These observations are in line with the results reported by Amigo-Benavent et al. [21].

## 4. Conclusion

In the present work, we showed that coffee extracts have antiproliferative and cytotoxic effects on breast cancer cells in 2D and 3D culture models. Surprisingly, coffee extracts do not affect viability on human epithelial breast cell lines (noncarcinogens). Our results suggest that green and roasted coffee bean extracts showed a strong bioactive capacity, promoting cell viability decrease, cell cycle alteration, cytostatic effect, mitochondrial morphology, and MMP alterations and clonogenic capacity loss.

These extracts of *Coffea canephora* have been characterized in a previous work, in which we found a total phenolic compounds with values of 2198.7 ± 71.0 (dark roasted freeze-dried) or 3051.1 ± 33.7 mg gallic acid equivalents/100 g (green freeze-dried) and a caffeine content between 0.1 and 0.3 g/100 g, respectively [9]. Although greater knowledge about the chemical composition of coffee extracts could help to understand the possible compounds responsible for the observed effect, many authors have investigated the effect of isolated compounds (caffeine, phenolic compounds, trigonelline, flavonoids, chlorogenic acid, caffeic acid, etc.), but could not attribute to a single compound in the bioactive properties of the complex mixture obtained when preparing coffee, because they use different extracts to those of a typical coffee prepared, or they enhance the extraction of bioactive compounds [10, 28, 30].

The results presented herein have far-reaching health relevance since coffee compounds could be used as chemopreventive and chemotherapeutic medicine which, in addition to their antioxidant activities and capacities, can also provide nutrition and contribute to preventing cancer development and progression. As suggested by other authors, a cancer diagnosis could be a stimulus for patients to make protective changes in health, and health professionals should consider this as a window of opportunity to educate patients about a healthy lifestyle [74]. Further, before these encouraging epidemiological observations can be confirmed and used as a sound basis for dietary advice, further research is needed on the bioavailability and pharmacokinetics of coffee [31]. In our experiments, we treated cells with concentrations equivalent to one cup of coffee (50 mL). However, we cannot immediately extrapolate these results to humans without considering the individual human metabolism, and further research (as in vivo experiments) is necessary to reach this conclusion.

## Data Availability

The data used to support the findings of this study are available from the corresponding author upon request.

## Supplementary Materials

Supplementary Materials Supplementary Figure 1: coffee extract effect on cell cancer viability. Human cancer cell lines (U2OS, HCT116, and T98G) were treated for 24 h with increasing concentrations of (a) green coffee or (b) dark coffee (0-5000 *μ*g/mL). (c) Inhibitory concentration 50% (IC50) on cancer cell line treated with green and dark coffee was determined and expressed as *μ*g/mL. Values are represented as mean ± SEM from three independent experiments. (d) Procaspase-3 and *α*-tubulin expression of MDA-231-MB and MCF 10A cells were treated with green coffee extracts and analyzed by western blot. (e) Procaspase-3/*α*tubulin western blot intensity were analyzed and quantified by ImageJ software. Values are mean ± SEM from two independent experiments (^∗∗∗^*p* < 0.0001 as determined by ANOVA).
