# Supplementary figures and images for: Antitumor Effects of Freeze-Dried Robusta Coffee (Coffea canephora) Extracts on Breast Cancer Cell Lines
Source: Oxid Med Cell Longev. 2021 May 18;2021:5572630. doi: 10.1155/2021/5572630 (PMC8154281; doi:10.1155/2021/5572630)

**A**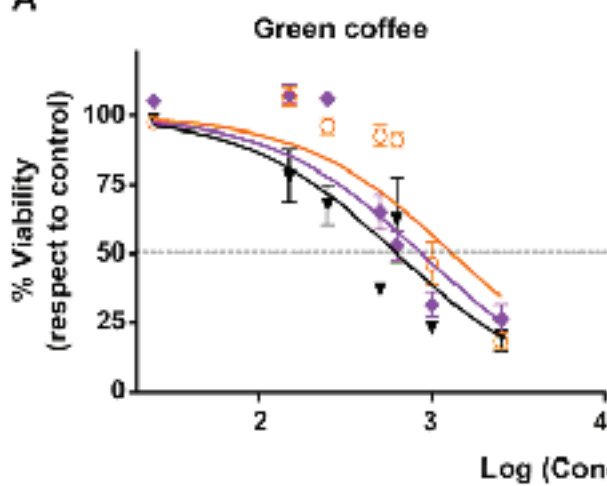**B**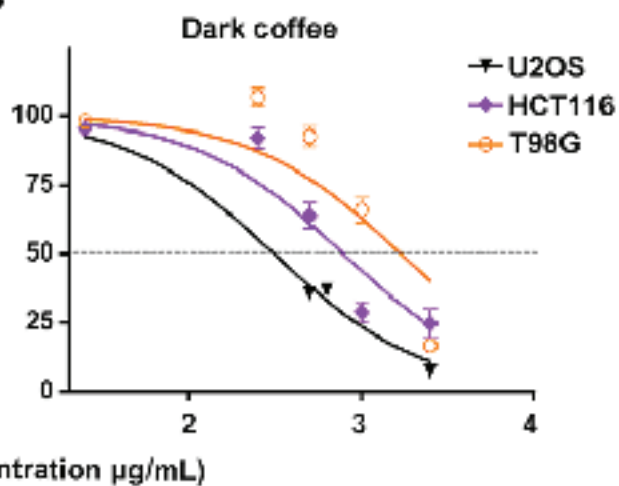**C**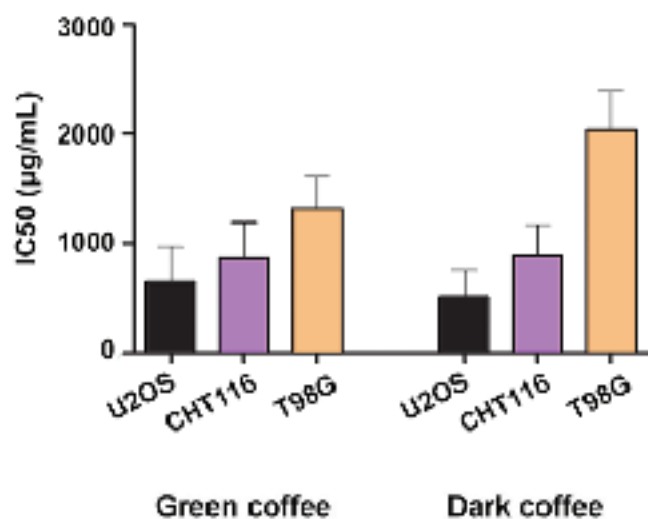**D**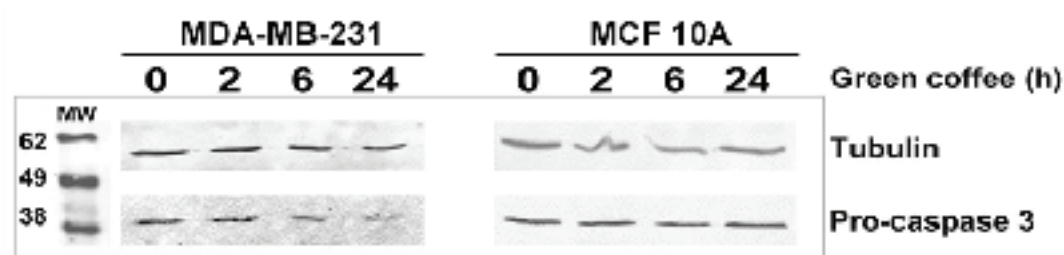**E**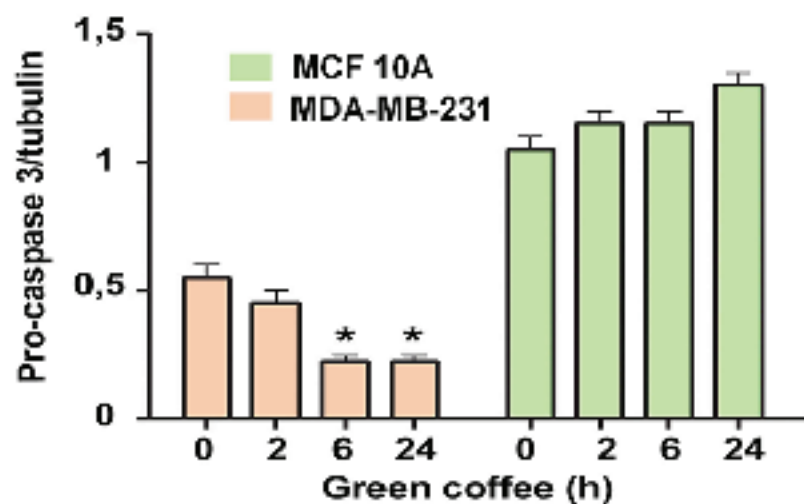

Supplement: Supplementary Materials — Supplementary Figure 1: coffee extract effect on cell cancer viability. Human cancer cell lines (U2OS, HCT116, and T98G) were treated for 24 h with increasing concentrations of (a) green coffee or (b) dark coffee (0-5000 μg/mL). (c) Inhibitory concentration 50% (IC50) on cancer cell line treated with green and dark coffee was determined and expressed as μg/mL. Values are represented as mean ± SEM from three independent experiments. (d) Procaspase-3 and α-tubulin expression of MDA-231-MB and MCF 10A cells were treated with green coffee extracts and analyzed by western blot. (e) Procaspase-3/αtubulin western blot intensity were analyzed and quantified by ImageJ software. Values are mean ± SEM from two independent experiments (∗∗∗p < 0.0001 as determined by ANOVA). [file 5572630.f1.pdf]
